# Elevated Levels of Circulating DNA in Cardiovascular Disease Patients: Metagenomic Profiling of Microbiome in the Circulation

**DOI:** 10.1371/journal.pone.0105221

**Published:** 2014-08-18

**Authors:** Vasudevan Dinakaran, Andiappan Rathinavel, Muthuirulan Pushpanathan, Ramamoorthy Sivakumar, Paramasamy Gunasekaran, Jeyaprakash Rajendhran

**Affiliations:** 1 Department of Genetics, Center for excellence in Genomic Sciences, School of Biological Sciences, Madurai Kamaraj University, Madurai, Tamilnadu, India; 2 Department of Cardiothoracic Surgery, Madurai Medical College & Government Rajaji Hospital, Madurai, Tamilnadu, India; Charité-University Medicine Berlin, Germany

## Abstract

Cardiovascular diseases (CVDs) are the leading cause of death worldwide. An expanding body of evidence supports the role of human microbiome in the establishment of CVDs and, this has gained much attention recently. This work was aimed to study the circulating human microbiome in CVD patients and healthy subjects. The levels of circulating cell free DNA (circDNA) was higher in CVD patients (n = 80) than in healthy controls (n = 40). More specifically, the relative levels of circulating bacterial DNA and the ratio of 16S rRNA/β-globin gene copy numbers were higher in the circulation of CVD patients than healthy individuals. In addition, we found a higher circulating microbial diversity in CVD patients (n = 3) in comparison to healthy individuals (n = 3) by deep shotgun sequencing. At the phylum level, we observed a dominance of Actinobacteria in CVD patients, followed by Proteobacteria, in contrast to that in healthy controls, where Proteobacteria was predominantly enriched, followed by Actinobacteria. The circulating virome in CVD patients was enriched with bacteriophages with a preponderance of *Propionibacterium* phages, followed by *Pseudomonas* phages and *Rhizobium* phages in contrast to that in healthy individuals, where a relatively greater abundance of eukaryotic viruses dominated by Lymphocystis virus (LCV) and Torque Teno viruses (TTV) was observed. Thus, the release of bacterial and viral DNA elements in the circulation could play a major role leading to elevated circDNA levels in CVD patients. The increased circDNA levels could be either the cause or consequence of CVD incidence, which needs to be explored further.

## Introduction

Cardiovascular diseases (CVD) remain the leading cause of death worldwide and continue to contribute significantly to disease induced morbidity and mortality [1]. Besides well-known risk factors, epidemiological and experimental evidences suggesting that infection is involved in the onset of CVD continues to accumulate [2], [3]. CVD can also be caused by transient bacteremia, which in turn is caused by trauma to mucosal surfaces densely populated with microbes. If untreated, bacteremia in these patients could progress into infective endocarditis. The microbial species causing transient bacteremia are largely dependent on the site exposed to trauma [4]. Several entry routes have been proposed in addition to physical interventions, for the ingress of bacteria into systemic circulation even in healthy individuals [5].

Many recent studies demonstrate an association between endotoxemia and atherosclerosis [6]. The organisms inducing transient bacteremia, nonetheless, are dependent on the microbial load and communities residing on the traumatized site. We recently described the presence of circulating microbiome and their dysbiosis in CVD patients compared with clinically healthy individuals [7]. The role of circulating cell-free DNA (circDNA), in particular, cell-free microbial DNA (microbiome) in the etiology or development of CVDs is unexplored, though the role of gut and oral microbiome in CVDs is well studied [8]–[10]. Circulating cell-free DNA is present in meager amounts in the plasma of healthy individuals. Of late, elevated levels of circDNA have been reported in various diseases, including cancer [11], stroke [12], trauma [13], autoimmune disorders [14] and pregnancy associated complications [15]. The concentration of circDNA has been reported to be increased in the circulation of CVD patients as well [16], [17]. Although the mechanism by which DNA is released into the circulation is not clearly defined, increased rates of cell death events either by apoptosis or necrosis are believed to be the major sources of circDNA [18].

However, circDNA might include DNA from human cells as well as from human microbiome. The passage of viable bacteria or bacterial components such as lipopolysaccharides (LPS) and peptidoglycan from the gastrointestinal tract (GI tract) through the epithelial mucosa is referred as bacterial translocation [19]. Similar to other bacterial components, bacterial DNA may also enter the circulation during translocation. In conjunction with this, more recently, studies on the translocation of viral DNA has gained much interest [20], [21]. Many biomarkers, including LPS and CD4+ counts have been used to quantify microbial/bacterial translocation in various conditions like cirrhosis, ascites, non-alcoholic steatohepatitis (NASH) and HIV/AIDS [22], [23]. Alternatively, 16S rRNA genes can be utilized to quantify bacterial DNA in the circulation.

Unlike other organs, blood was originally presumed to be sterile, and microbes were thought to occur in circulation only in sepsis cases. But, in recent decades, presence of bacterial 16S rRNA genes has been reported in the circulation of healthy individuals [24], [25]. Adding to this knowledge are the recent reports on identification of viruses in the circulation of asymptomatic humans [26], [27]. Nevertheless, the source of bacterial and viral DNA elements in the circulation of healthy individuals remains elusive. Thus, quite contrary to the common misconception that circulating blood is sterile in healthy individuals; the blood too transiently harbors a microbiome, which is likely to be altered upon disease incidence.

Viral communities have been characterised from different human anatomical sites, including gut, skin and blood [28]–[30]. Human circulation harbours heterogeneous viral flora, and most of these viruses are part of normal flora and rarely cause disease in healthy population [30], [31]. Unlike bacteria, viruses do not carry any conserved genes for prompt detection. Albeit determining whether a virus is a typical commensal or an intrusive pathogen has direct clinical applications, this continues to be a challenging process.

We performed deep shotgun sequencing of plasma samples from selected CVD patients and healthy controls and profiled the taxonomic and functional distribution of bacterial and viral elements in the circulation. In recent years, besides studies on the human microbiome, viral metagenomics has become an established method to study the human virome [32], [33]. With the advent of next generation sequencing (NGS)-based metagenomics, large-scale detection and profiling of bacterial and viral elements in several anatomical sites of the human body has become more feasible with greater sensitivity [34], [35]. An overall progress in understanding the etiology of many diseases has been impeded primarily because of problems in detecting potential pathogens as well as in relating the prevalence of a particular bacteria or virus to specific signs of infection. Nevertheless, NGS technologies are developing at a rapid pace to identify unknown agents and an extensive range of computational tools are currently available for microbiome and virome analysis.

More recently, metagenomic shotgun sequencing has been employed widely for clinical samples. For example, shotgun sequencing has been employed to sequence the plasma of HIV patients to detect bacterial and viral elements in circulation [36]. The microbial/viral ecology of pathogenicity remains poorly understood in CVDs. Microbes are believed to play a part in the pathogenesis of CVD; per contra, no specific etiological agents have been identified so far in most of the CVDs. More precisely, to the best of our knowledge, the presence of viruses and their association with bacteria in CVD patients has not been investigated yet. So, we employed shotgun sequencing to compare microbial and viral elements in selected CVD patients and clinically healthy subjects to profile the genes implicated in the pathogenesis of CVD.

Hence, in the present study, we quantified the total circDNA in CVD patients and healthy individuals using a sensitive picogreen fluorescence assay. The relative levels of bacterial and human DNA elements were determined by the copy numbers of 16S rRNA and β-globin genes respectively. Additionally, the ratio of 16S rRNA/β-globin gene copy numbers in CVD patients and healthy individuals were compared. Further, bacterial and viral elements and their genes involved in pathogenesis were comparatively profiled in CVD patients and apparently healthy individuals by barcoded shotgun sequencing.

## Materials and Methods

### Ethics Statement

The participants provided both written and verbal informed consent to participate in this study. The written informed consent was obtained from the participants by explaining them the objective and purpose of the study in their respective mother tongue (regional language). This consent procedure was approved by the Internal Research Review Board & Ethical Clearance Committee of Madurai Kamaraj University.

The written informed consent contained the following details:

- Clear explanation of the objective and purpose of the study in their respective mother tongue (regional language).
- Informed written consent obtained from the guardians on behalf of the minors/children enrolled in our study.
- Assurance to the participants that the collected samples will be used only for research purpose and not for any other use.
- The researchers have rights to disclose the results by publishing them in scientific journals keeping in view its use for the society and scientific community.
- The results will not be disclosed by any other means.

### Study population

The blood samples were collected from 80 CVD patients attending the out-patient section of Government Rajaji hospital, Madurai, Tamil Nadu, India from the period of July 2010 to March 2011. Patients were selected based on their clinical presentations, ECG and ECHO patterns, clinical history and those who were not under antibiotic therapy for a minimum of four weeks before the date of collection of blood sample. Moreover, none of the patients were suffering from any inflammatory disease or other co-morbidity, which might provoke bacteremia. The study samples include 24 valvular heart disease (VHD) patients, 30 ischemic heart disease (IHD) patients, 26 congenital heart disease (CHD) patients and 40 healthy blood donors.

### Extraction of plasma DNA

Peripheral blood samples were collected aseptically by venipuncture in EDTA containing tubes from CVD patients and healthy subjects. Plasma was separated by centrifugation for 10 min at 6000×g under aseptic conditions. The separated plasma was transferred into a 1.5 ml sterile polypropylene tube without disturbing the buffy coat layer. The newly separated aliquot was centrifuged for another 10 min at 16,000×g. The upper part of the plasma was then transferred into a clear sterile tube and frozen at −20°C prior to DNA extraction. CircDNA was extracted from 200 µl of plasma using the QIAamp DNA blood mini kit (Qiagen, Hilden, Germany).

### Quantification of circDNA in plasma

CircDNA concentration in plasma was determined using Quant-iT PicoGreen dsDNA assay kit (Invitrogen Molecular Probes Inc., Eugene, OR, USA). PicoGreen assay is a highly sensitive tool to determine even very low concentrations of DNA (upto picogram level) [37]. Plasma DNA samples were mixed with the PicoGreen dye in 1∶1 ratio in a black 96-well microtiter plate (Nunc, Denmark), and fluorescence was measured using a SpectraMax M2^e^ Microplate Reader (Molecular Devices, Sunnyvale, CA, USA) with an excitation wavelength of 485 nm and an emission/detection wavelength of 535 nm. Lambda phage DNA was used as the standard.

### Quantification of circulating 16S rRNA and β-globin genes in plasma

Circulating 16S rRNA and β-globin genes were quantitated by real-time PCR using the CFX96 System (BioRad, CA, USA). The laboratory and equipments used for this study were dedicated to cell-free DNA extraction and quantitation to avoid contamination of the samples with DNA from other sources. An *Escherichia coli* 16S rRNA gene fragment cloned in pTZ57R/T (Thermo Fisher Scientific, Waltham, MA, USA) was used as the standard for quantification of 16S rRNA gene. Similarly, a human β-globin gene fragment cloned in pTZ57R/T was used as the standard for quantification of β-globin gene. Sterile nuclease free water was used as no template control (NTC).

The primers used for the quantitative PCR assay are listed in Table S2 in Supporting Information S1. The assays were performed using Maxima SYBR Green/NOROX qPCR master mix (2X) (Thermo Scientific). The SYBR Green master mix was pretreated with DNase I for 2 hours at 37°C to remove DNA contamination in the reagents. DNase I was then heat inactivated at 70°C for 10min prior to the assay. Quantitative PCR was carried out in a final volume of 25 µl containing 12.5 µl of 2X master mix, 300 nM of respective primers and 5 µl of plasma DNA, employing the following thermal cycling conditions: 95°C for 10 min; 40 cycles of 95°C for 15 s, and 60°C for 1 min [38], [39]. All experiments were carried out in triplicates.

### Metagenomic shotgun sequencing of circDNA of CVD patients and healthy individuals

Selected plasma DNA samples from 3 CVD patients (males  = 3; females  = 0) and 3 healthy subjects (m = 2; f = 1) were subjected to Shotgun sequencing using the Ion Torrent Personal Genome Machine (PGM) (Life Technologies, California, USA). The CVD patients were diagnosed with Bicuspid aortic valvular heart disease (BCAV) (sample ID: CVD008), Partial anomalous pulmonary venous connection (PAPVC) (sample ID: CVD010) and coronary artery disease (CAD) (sample ID: CVD014) respectively. The baseline characteristics of CVD patients and healthy participants used for the Plasma DNA sequencing study are described in Table S3 in Supporting Information S1.

### Whole genome amplification of circDNA

The yield of plasma DNA extracted from 200 µl of plasma was approximately, ≤10 ng/µl. To increase the yield of extracted DNA, the plasma DNA samples were amplified using REPLI-g Mini kit (Qiagen, Hilden, Germany) employing multiple displacement amplification (MDA) procedure, which uniformly amplifies the entire genome. After MDA, a concentration of >10 ng/µl DNA was obtained in each sample. The amplified DNA for each sample was pooled from three separate reactions to minimize bias.

### Bar-coded shotgun sequencing of circDNA

The amplified plasma DNA samples were fragmented individually using Ion Shear Plus Reagents kit (Life Technologies) to obtain a desired size range of 100–600 bp with a maximum concentration around the 200 bp region. Barcoded A and P1 adapters were ligated to the termini of PCR products and the ligated fragments were size selected on 2% E-Gel (Invitrogen). After quantification of individual libraries using a bioanalyzer (MultiNA, Shimadzu), the libraries were pooled in equimolar concentrations and were subjected to amplification. Emulsion PCR was carried out using the Ion Xpress Template kit V2.0 (Life Technologies) as per manufacturer's instructions on Ion One Touch template preparation platform. Charged template positive ion sphere particles (ISPs) were enriched from the pool of resulting ISPs using Ion One Touch enrichment system (ES). Charged ISPs were then loaded on to an Ion 316 chip as per manufacturer's recommendations and sequencing of the pooled libraries was carried out on the Ion Torrent PGM using the Ion Sequencing 200 kit (Life Technologies). All the samples were sequenced in triplicates.

### Processing of Sequence reads

Generated raw reads were pre-processed for adapter removal while preserving barcode sequences. Quality trimming of reads was achieved using Beverley filter algorithm on Torrent suite v2.2. Processed sequence reads were downloaded as *sff* files from Torrent server and converted into fasta, quality and flow files using Mothur package [40]. The *de novo* assembly of sequences was done using MIRA (mimicking intelligent read assembly) version 3.4 [41].

The metagenomics RAST (MG-RAST) server version 3.2 was used for further downstream analyses using SEED database as reference [42]. The CloVR-Metagenomics version 1.0 pipeline was used for taxonomical annotation of the bacterial communities [43]. For the overall phylogenetic designation of sequence reads at phylum level- default parameters were 80% similarity over 100 bases at 1e^−5^. The Data Intensive Academic Grid (DIAG) computational cloud was used in conjunction with the CloVR-Metagenomics automated pipeline using the “Open Read Frames (ORFs)” option to execute computationally intensive tasks. The WebMGA server [44], which uses the COGs database, was employed for functional annotation of bacterial communities to identify virulence genes, toxins, antibiotic resistance genes and genes essential for microbes to evade host immune surveillance mechanisms. The accession numbers for the sample-specific flowgram files submitted to MG-RAST server starts from 4529237.3 through 4529242.3 and were assigned an MG-RAST project ID (mgp5220).

The MetaVir web server, which uses the GAAS tool and PFAM database and the Virome pipeline, which uses two unique sequence databases as core, *viz.*, the UniRef 100 Plus (UniRef100P) pipeline and Metagenomes On-Line (MGOL) database were employed for the taxonomical annotation of viral communities [45], [46]. The MetaVir server uses the taxonomic composition tool, which gives taxonomic affiliations of the viral sequence reads. This taxonomic affiliation is gathered with the GAAS tool [47], especially developed to deal with virome sequences. More precisely, the viral sequence reads are compared to PFAM database, and the number of taxonomic affiliations is normalized by the genome length of the associated viral species in order to compare the different number of virion-like particles initially present in the sample.

### Statistical analysis and evaluation

Quantification data were expressed as median (inter-quartile range, IQR). Non-parametric statistical tools (Mann-Whitney *U* test and Kruskal-Wallis test) were applied to compare quantification data. Pair-wise comparison was achieved using Mann-Whitney *U* test, while categorical variables were compared using Kruskal-Wallis test. A *p* value of <0.05 was considered statistically significant. Statistical analyses of the quantification data were performed using the R software package (version 2.15.2).

Receivers operating characteristic (ROC) curve was constructed to estimate an optimal cut-off point or criterion value for the use of both 16S rRNA and β-globin copy numbers to discriminate VHD, IHD and CHD patients from healthy individuals. Sensitivity, specificity and 95% confidence interval (CI) values were computed using the cutoff point determined by ROC analysis. Accuracy of the test was determined using the area under the curve (AUC) in ROC analysis. Statistical significance of differences between areas under two or more ROC curves for VHD, IHD, CHD patients and control groups was determined using MedCalc software (version 12.4.0, MariaKerke, Belgium) based on the method described by Hanley and McNeil [48]. In addition, multiple regression analysis (multivariate model) was performed to assess the independent relation between circulating DNA and cardiovascular status using MedCalc software (version 12.4.0, MariaKerke, Belgium). Statistical evaluation of taxonomical annotation of bacterial communities was performed using R software package inbuilt in the CloVR-pipeline. While, statistical evaluation of taxonomical annotation of the viral communities were performed using Rcmdr package in the R software package (version 2.15.2).

## Results

### Study population

The baseline characteristics of the CVD patients (n = 80) and the healthy control subjects (n = 40) are listed in Table S1 in Supporting Information S1. Based on the clinical manifestations and clinical history, CVD patients were classified into three groups, *viz.*, valvular heart disease (VHD), ischemic heart disease (IHD) and congenital heart disease (CHD) patients. VHD patients were treated with antibiotics, cardiac drugs, analgesics and anti-anxiety drugs in a patient specific manner and some VHD patients were convalescing from valvular replacement surgery. Individuals of the IHD group were treated with cardiac drugs, anti-platelet drugs, and anti-anxiety drugs. Depending on the severity of the disease, patients were advised to undergo graft surgeries. In particular, most of the coronary artery disease (CAD) patients were convalescing from Coronary Artery Bypass Graft (CABG) surgery and Cardiopulmonary Bypass (CPB) surgery. Patients suffering from congenital heart disease (CHD) were mostly offered palliative care or surgery in a case-by-case manner and some patients have undergone intra cardiac repair.

### Elevated levels of circDNA in the plasma of CVD patients

**Figure 1:**
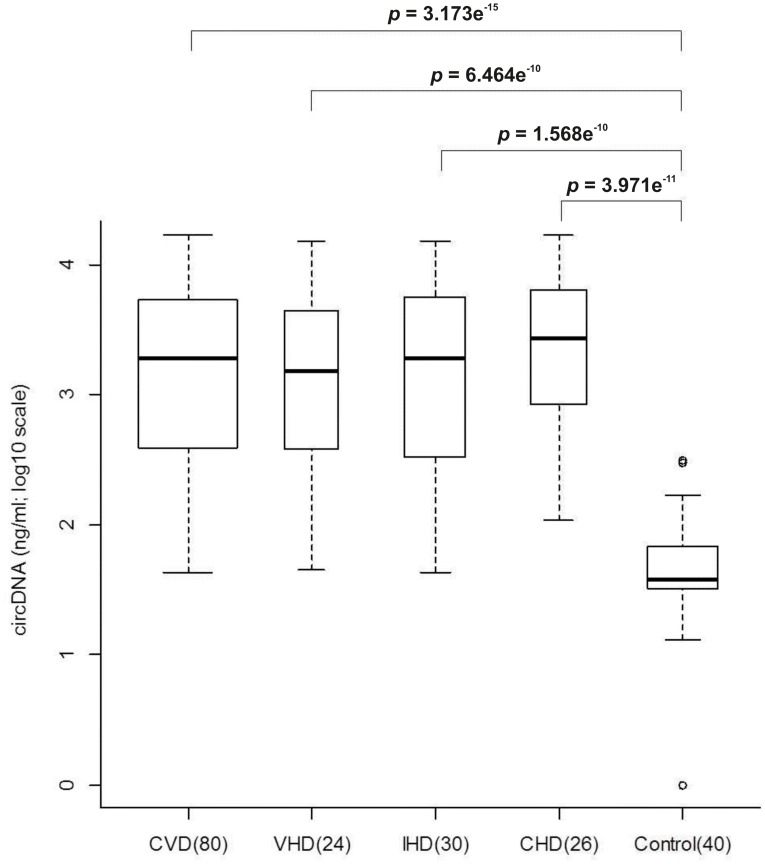
Levels of circDNA in the plasma of CVD patients and healthy control samples (in Log10 scale).

The concentration of circDNA in plasma was determined by Picogreen fluorescence assay. The circDNA level in the plasma of CVD patients (median, 1925.64 ng/ml; IQR = 391.83, 5395.92) was significantly higher (*p* = 3.173e^−15^; Kruskal-Wallis test) than the healthy individuals (median, 37.85 ng/ml; IQR = 32.80, 69.38). The median concentration (IQR) of circDNA in VHD, IHD and CHD patients were 1548.99 ng/ml (383.99, 4476.41), 1898.66 ng/ml (335.26, 5613.19) and 2965.81 ng/ml (844.06, 6474.97) respectively. Thus, the mean circDNA concentrations in VHD, IHD and CHD patients were 41-fold, 50-fold and 71-fold higher than the control samples respectively. The differences in the copy numbers of 16S rRNA genes were statistically (Mann-Whitney *U* test) significant (Fig 1).

### Increased ratio of circulating 16S rRNA/β-globin copy number in CVD patients

The copy number of bacterial 16S rRNA gene and human β-globin gene were determined by the respective quantitative PCR assays. All calibration curves showed linearity over the entire quantification range with a correlation coefficient (R^2^) value of ∼0.99, a mean slope of ∼−3.3, and a PCR efficiency of >95%, indicating a precise log-linear relationship. No fluorescence signal was detected from no template controls (NTC), showing the lack of contamination in quantitative PCR reagents.

**Figure 2:**
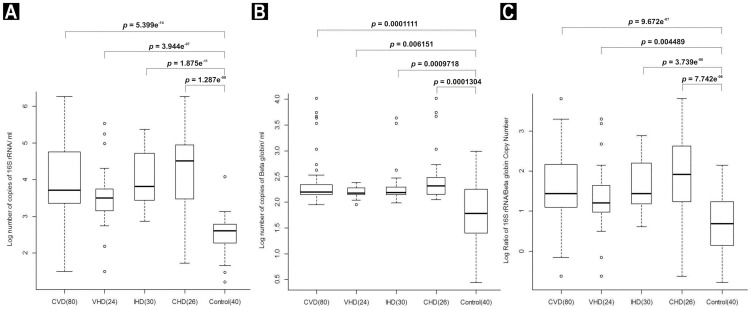
Number of copies of 16S rRNA gene (A), number of copies of β-globin gene (B) and the ratio of number of copies of 16S rRNA to β-globin (C) in the plasma of CVD patients and healthy control samples (in Log10 Scale).

The 16S rRNA gene copy number in the plasma of CVD patients (median = 5289.87; IQR = 2293.26, 57331.51) was significantly higher (*p* = 5.399e^−14^; Kruskal-Wallis test) than the healthy individuals (median = 411.73; IQR = 186.62, 617.16). The median copy number (IQR) of 16S rRNA genes/ml in the plasma of VHD, IHD and CHD patients were 3143.78 (1409.86, 5644.49), 6603.15 (2757.23, 52012.09) and 32803.08 (3040.42, 90134.59), respectively. Thus, the mean 16S rRNA gene copy number in the circulation of VHD, IHD and CHD patients were nearly 8-fold, 16-fold and 80-fold higher than the healthy control samples respectively. The differences in the copy numbers of 16S rRNA genes were statistically (Mann-Whitney *U* test) significant (Fig 2A).

Similarly, the copy number of β-globin gene in the plasma of CVD patients (median = 158.89; IQR = 141.35, 218.44) was also significantly higher (*p* = 0.0001111; Kruskal-Wallis test) than the healthy individuals (median = 60.19; IQR = 25.05, 178.88). The median copy number (IQR) of β-globin gene/ml of plasma in VHD, IHD and CHD patients were 152.07 (140.78, 188), 155.44 (142.37, 196.43) and 208.26 (144.48, 302.46) respectively. Thus, the median β-globin gene copy numbers in the circulation of VHD and IHD patients were more than two-fold higher than the healthy control samples. Whereas, the median β-globin gene copy number in the circulation of CHD patients was more than three-fold higher than the healthy control samples. The differences in the copy numbers of β-globin genes were statistically (Mann-Whitney *U* test) significant (Fig 2B).

In order to study the relative contributions of bacterial and human DNA in the entire circDNA concentration, we calculated the ratio of copy numbers of 16S rRNA/β-globin genes in both the test as well as control samples. The ratio of 16S rRNA/β-globin copy numbers in the plasma of CVD patients (median = 27.29; IQR = 12.71, 147.72) was significantly higher (*p* = 9.672e^−07^; Kruskal-Wallis test) than the healthy individuals (median = 4.78; IQR = 1.38, 16.95). The 16S rRNA/β-globin gene copy number ratio in VHD, IHD and CHD patients were 15.86 (9.41, 44.5), 27.29 (15.13, 159.72) and 83.81(17.03, 428.61) respectively. Thus, the mean ratio in VHD, IHD and CHD patients were more than three-fold, five-fold and 17-fold higher than the healthy control samples respectively and the differences were statistically (Mann-Whitney *U* test) significant (Fig 2C).

### Sensitivity and specificity of circDNA as a candidate marker

The sensitivity and specificity of circDNA levels, to be considered a candidate diagnostic marker for CVDs, was evaluated using the Receiver Operating Characteristic Curve (ROC) analysis. The area under the curve (AUCROC) for the circDNA concentrations was 0.951 (95% CI, 0.864–0.990; *p*<0.0001) and the cut-off point or criterion value for circDNA for discriminating VHD patients and healthy individuals was 313.98 ng/ml (87% sensitivity and 100% specificity). The AUCROC for 16S rRNA gene was 0.630 (95% CI, 0.498–0.750; *p*<0.0001), whereas the AUCROC value for β-globin gene was 0.724 (95% CI, 0.596–0.830; *p*<0.0001). The cut-off points for circulating 16S rRNA and β-globin genes for discriminating VHD patients from healthy individuals were 937 copies/ml (87% sensitivity and 94.9% specificity) and 105 copies/ml (95.7% sensitivity and 61.5% specificity), respectively.

The AUCROC for the circDNA concentrations was 0.925 (95% CI, 0.835–0.975; *p*<0.0001) and the cut-off point for circDNA for discriminating IHD patients and healthy individuals was 313.98 ng/ml (93.1% sensitivity and 82.1% specificity). The AUCROC for 16S rRNA gene was 0.966 (95% CI, 0.892–0.995; *p*<0.0001), whereas the AUCROC value for β-globin gene was 0.746 (95% CI, 0.626–0.844; *p*<0.0001). The cut-off point for circulating 16S rRNA and β-globin genes for discriminating IHD patients from healthy individuals were 937 copies/ml (96.6% sensitivity and 94.9% specificity) and 105 copies/ml (96.6% sensitivity and 61.5% specificity), respectively.

**Figure 3:**
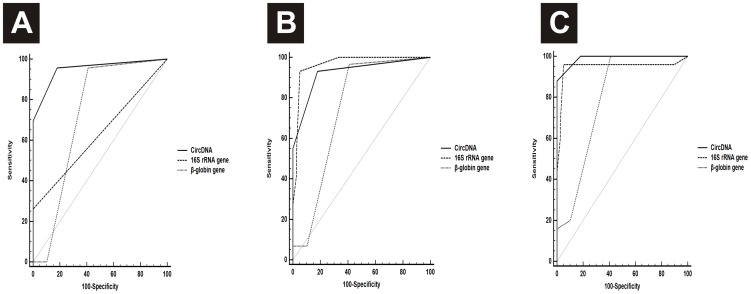
Receiver operating characteristic (ROC) curve for plasma 16S rRNA gene levels, β-globin gene levels and circDNA levels of (A) Valvular Heart disease (VHD), (B) Ischemic Heart disease (IHD) and (C) Congenital Heart disease (CHD) patients.

The AUCROC for the circDNA concentrations was 0.989 (95% CI, 0.924–1.000; *p*<0.0001) and the cut-off point for circDNA for discriminating CHD patients and healthy individuals was 313.98 ng/ml (96% sensitivity and 100% specificity). The AUCROC for 16S rRNA gene was 0.947 (95% CI, 0.861–0.988; *p*<0.0001), whereas the AUCROC value for β-globin gene was 0.793 (95% CI, 0.673–0.884; *p*<0.0001). The cut-off point for circulating 16S rRNA and β-globin genes for discriminating CHD patients from healthy individuals were 1354 copies/ml (92% sensitivity and 97.4% specificity) and 105 copies/ml (100% sensitivity and 61.5% specificity), respectively (Fig 3). Except VHD patients, where, the AUC for 16S rRNA gene copy number was less than 0.7, the AUC for circDNA and 16S rRNA gene copy number for all the CVD patients were >0.9, and hence these parameters can be considered to discriminate CVD patients from healthy individuals.

### Relation between circDNA and cardiovascular status

The independent relation between circDNA, 16S rRNA, β-globin and cardiovascular status in VHD, IHD and CHD patients was assessed by multiple regression analysis between these levels and the four parameters indicating cardiac status *viz.*, Blood pressure, Heart rate, C-reactive protein (CRP) levels and Procalcitonin (PCT) levels. The significance level for the F-test was <0.05 in all the cases, indicating that the multiple correlation co-efficient is statistically significant and all three variables (circDNA concentration, 16S rRNA copy number, β-globin copy number) are statistically dependent on the cardiac status of the patients.

The statistical significance level between circDNA, 16S rRNA, β-globin and cardiovascular status in VHD patients was 0.0492, 0.0102 and <0.0001 respectively, in IHD patients was 0.0246, 0.0004 and 0.0497 and in CHD patients was 0.0008, <0.0001 and 0.0362 respectively. This demonstrates that circDNA, 16S rRNA and β-globin are dependent on the cardiovascular status of the patients.

### Comparison of microbiome in the circulation of CVD patients and healthy individuals

**Figure 4:**
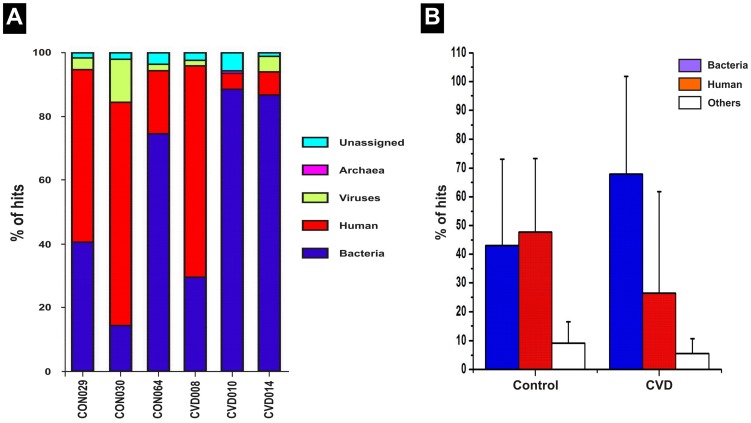
Relative abundance of bacterial, human, viral and archeal signatures in human plasma as profiled by metagenome shotgun sequencing.

We employed metagenomic shotgun sequencing of plasma DNA to reveal plausible differences in the circulating microbiome of CVD (n = 3) patients and healthy individuals (n = 3). After demultiplexing and quality control, a total of 1,729,603 sequence raw reads (258,111,408 bp) with an average read length of 149 bp were obtained. However, the number of sequence raw reads obtained for CVD plasma samples was far higher than that obtained for the control plasma samples. The sequence throughput for CVD samples was CVD008: 449,185 reads (55,417,060 bp); CVD010: 568,109 reads (95,670,252 bp) and CVD014: 216,817 reads (32,840,290 bp) while the sequence throughput for control samples was CON029: 135,900 reads (22,250,079 bp); CON030: 169,011 reads (24,576,839 bp) and CON064: 190,581 reads (27,356,888 bp) respectively. Based on the sequence analysis using MG-RAST server, 68% of reads from the circDNA of CVD samples and 43% of reads from control samples were assigned to the domain bacteria using SEED database as reference. In contrast, only 27% of the reads from CVD samples were assigned to human as compared to 48% of the sequences in healthy individuals. Thus, CVD patients had a higher concentration of bacterial DNA elements in the circulation than human DNA when compared to healthy individuals (Fig 4). These results were concordant with the data obtained by quantification of bacterial and human DNA elements using quantitative PCR. Viral and archeal DNA elements formed only a sparingly small fraction of the sequence reads. A higher proportion of archaeal DNA elements were observed in CVD patients (0.14%) than healthy controls (0.01%). On the contrary, a lesser fraction of viral DNA elements were observed in CVD patients (2.2%) than healthy individuals (6.7%).

### Differences in the distribution of bacterial signatures in the circulation of CVD patients and healthy individuals

**Figure 5:**
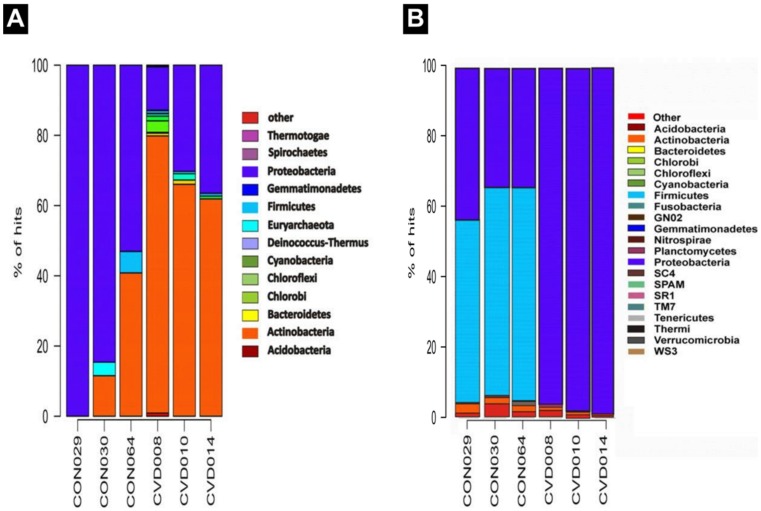
Comparison of bacterial compositional summary of control and CVD samples by deep shotgun sequencing (A) and amplicon sequencing (earlier study) (B). (A) Major compositional differences include (i) higher frequency of phylum actinobacteria than phylum proteobacteria among CVD samples (ii) higher frequency of Proteobacteria than Actinobacteria among healthy control samples (iii) negligible presence/complete absence of the Phylum actinobacteria in the control sample CON029. (B) Relative distribution of bacterial phyla obseveved by amplicon sequencing. Proteobacteria and Firmicutes were dominant in control samples and only Proteobacteria was dominant among CVD samples [7].

**Figure 6:**
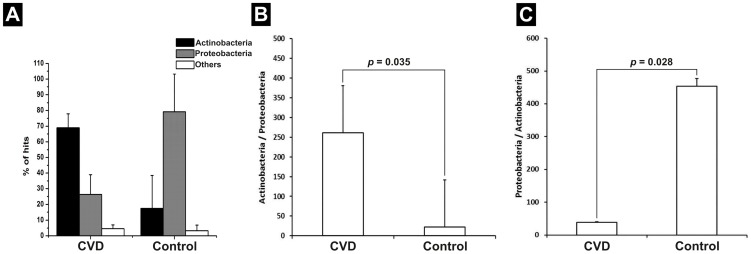
The ratio of Actinobacteria: Proteobacteria in CVD patients and healthy controls. (**A**) Relative occurrence of Actinobacteria and Proteobacteria in the circulating microbiome of CVD patients and healthy controls. An increase in Actinobacterial population coupled with a parallel reduction in Proteobacterial population in CVD patients is discernible. (**B**) Increase in the ratio of Actinobacteria / Proteobacteria (*p* = 0.035) in CVD patients compared to controls. (**C**) Increase in the ratio of Proteobacteria / Actinobacteria (*p* = 0.028) in controls compared to CVD patients.

The taxonomical annotation of the sequences was done using the CloVR- Metagenomics pipeline [43]. The taxonomic heat map provides comparative metagenomics summaries that encapsulate differences between samples (Fig 5). CVD patients had a higher bacterial diversity when compared to healthy individuals. Taxonomic assignment at the phylum level revealed that Actinobacteria and Proteobacteria were the two major phyla represented in both control and CVD samples, followed by a smaller fraction of Firmicutes (Fig 5). Proteobacterial population was significantly larger (*p* = 0.01; Mann-Whitney *U* test) than that of Actinobacteria in control samples. However, in CVD samples, a higher population of Actinobacteria (*p* = 0; Mann-Whitney *U* test) and comparatively lesser proportion of Proteobacteria was observed (Fig 6A), resulting in a significantly increased (*p* = 0.035; Mann-Whitney *U* test) Actinobacteria: Proteobacteria ratio (Fig 6B).

At a higher taxonomic resolution, our study identified a significant increase in the population of family Propionibacteriaceae of Actinobacteria with a parallel reduction in the population of family Pseudomonadaceae and class alpha proteobacteria in CVD patients. This was in contrary to the results obtained in our previous study [7] based on 16S rRNA gene amplicon sequencing.

**Figure 7:**
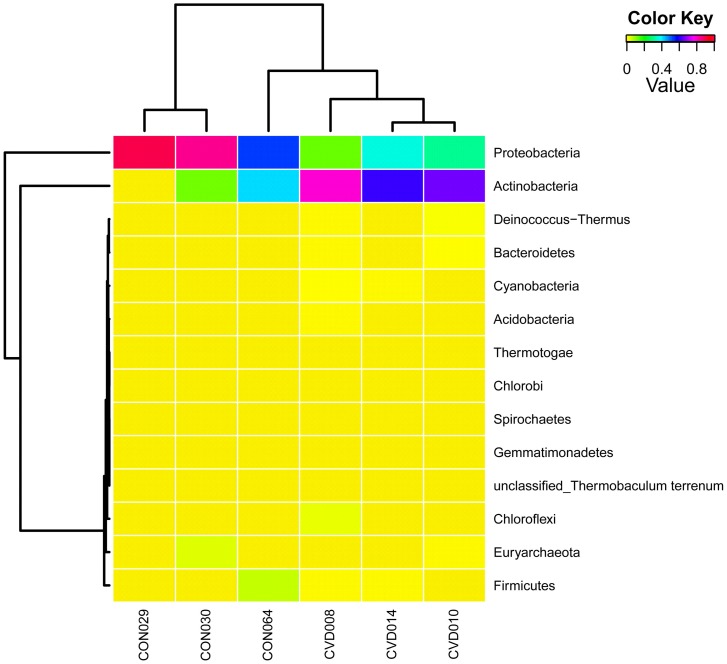
OTU heatmap displaying the distribution of OTUs from all 6 samples. The heatmap highlighting the distribution of CVD and healthy sample specific taxonomic lineage are indicated. Phyla Proteobacteria and Actinobacteria are dominant both in test and control samples. Phylum Actinobacteria is dominant among test samples while Phylum Proteobacteria is dominant among control samples.

Firmicutes and Euryarcheota were present at basal levels (≤2%) in both CVD and control samples with a marginally higher count in control samples. However, some members of the phylum Firmicutes, *viz.*, *Streptococcus*, *Bacillus*, *Acidaminococcus*, *Micrococcus*, etc. were detected only in CVD patient samples. In addition, populations of the phyla Bacteroidetes, Chlorobi, Chloroflexi, Cyanobacteria, Acidobacteria, Spirochetes, Deinococcus-Thermus, Gemmatimonadetes and Thermotogae were found in a very small fraction in CVD samples and not in healthy samples, reflecting a larger bacterial diversity in CVD patients in comparison to healthy individuals (Fig 7).

Among the members of phylum Actinobacteria, family Propionibacteriaceae was found in greater numbers in CVD samples in comparison with healthy samples. Other members of the phylum Actinobacteria, *viz.*, *Corynebacterium*, *Rhodococcus*, *Mycobacterium*, *Bifidobacterium*, *Brachybacterium*, *Clavibacter*, *Nocardia, Kocuria*, *Mobiluncus*, *Arthrobacter*, *Actinobacillus*, *Acinetobacter*, *Streptomyces*, *Cellulomonas*, *Leifsonia*, etc. were also found in greater numbers in CVD samples than healthy samples. The orders Rhizobiales, Rhodobacterales and Enterobacteriales, populated majorly by genera *Sinorhizobium*, *Myxobacterium*, *Escherichia*, *Bradyrhizobium* and *Methylobacterium* were detected in higher levels in CVD samples.

Bacterial communities in control samples were predominantly inhabited by the phylum Proteobacteria comprising largely of genera *Pseudomonas*, *Rhodopseudomonas*, *Escherichia*, *Shigella*, *Paracoccus*, etc. followed by phylum Actinobacteria with a preponderance of the genera *Propionibacterium* and *Corynebacterium*. Possible source(s) of these bacterial elements in the circulation of healthy individuals remains obscure.

The genus *Propionibacterium* was predominant among CVD patients samples while, *Pseudomonas* was the dominant genus among healthy controls. Phylum Euryarchaeota, comprising primarily of the genus *Thermococcus* was seen in both test and control samples. The conclusions drawn are based on the mean relative abundances of bacterial taxa in both disease and control groups in replicates, although slight variations in samples within each group were observed.

### Differences in the distribution of virome in the circulation of CVD and control groups

**Figure 8:**
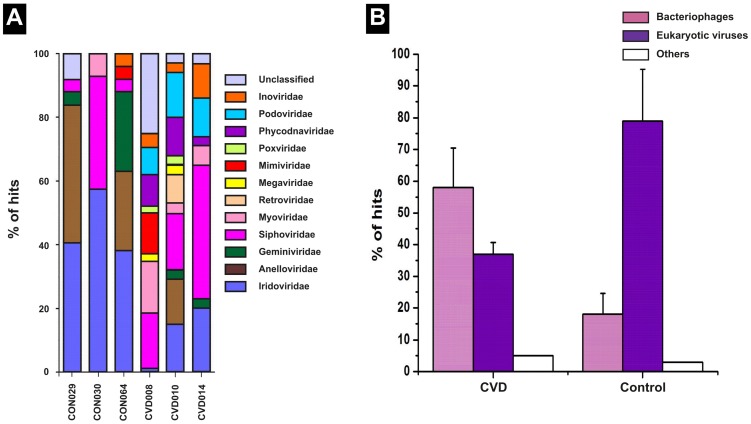
Comparison of the viral compositional summary of control and CVD samples by deep shotgun sequencing. (**A**) Major compositional differences include (i) relatively higher abundance of families Siphoviridae, Myoviridae, Podoviridae and Inoviridae among CVD samples than healthy individuals (ii) relatively higher abundance of families Mimiviridae, Poxviridae, Phycodnaviridae, Iridoviridae, Anelloviridae and Geminiviridae among healthy control samples (iii) negligible presence of the family Retroviridae in all the samples. (**B**) Relative occurrence of Bacteriophages and Eukaryotic viruses in the circulating microbiome of CVD patients and healthy controls. An increase in the population of Bacteriophages coupled with a parallel reduction in the population of Eukaryotic viruses in CVD patients is discernible.

The MetaVir tool and Virome pipeline were used to examine the genetic diversity of viral sequences [44], [45]. Viral elements made up to 4.3% and 2.3% of the overall contigs from control and CVD samples respectively. Contigs containing sequences of viral origin were further divided into viral species based on their closest homolog. We observed a huge diversity of viral elements among CVD samples than among control samples (Fig 8A). Most of the annotated viral communities were bacteriophages present in parallel with the corresponding bacterial hosts in the circulation. In CVD patients, virome was predominantly populated by bacteriophages which contributed 63% of the total viral sequences. In contrast, among healthy control samples, virome was dominated by eukaryotic viral elements which formed up to 79% of the viral sequences (Fig 8B). More specifically, phages of *Propionibacterium*, *Pseudomonas* and *Rhizobium* constituted the major fraction of bacteriophages in the circulation of CVD patients. The abundance of these bacteriophages is consistent with the corresponding bacterial sequences in the circulation. In healthy control samples, phages of *Pseudomonas* and *Rhizhobium* were seen in comparatively lesser fractions, correlating with the proportion of Proteobacterial sequences in these samples.

The taxonomic composition and abundance of viral elements in the six samples at the family level are summarized in Fig 8A. Among CVD samples, bacteriophages of the families Siphoviridae (33%) and Podoviridae (11%) contributed a larger proportion of viral sequences. The wide prevalence of these phage families in all three CVD samples likely reflects the common infection status of phages in the blood microbiome of CVD patients. In contrast, among control samples, eukaryotic viral elements belonging to families Iridoviridae (45%) and Anelloviridae (23%) accounted for most of the viral hits.

At a higher taxonomic resolution, our study revealed the sequences of 103 different viral species, including 93 different viral species from CVD patients and 17 different viral species from healthy controls respectively. Of note, we observed 86 different viral species exclusively from CVD samples and nine different viral species solely from control samples.

## Discussion

We humans along with our microbial dwellers are considered ‘supra-organisms’ with enormous metabolic diversity [49]. The human body harbours many local microorganisms, with diverse communities at different anatomical sites [50]. The blood, like other organs of the human body, has been shown to harbor its own microbial load even in healthy individuals [24]. Elevated circDNA levels in CVD patients have been reported earlier. Shimony et al. [51] reported nearly two-fold higher circDNA concentrations in patients with myocardial infarction than in healthy controls. Similarly, in another study, circDNA levels were reported to be higher in the non-survival group than survival-to-discharge group of cardiac arrest patients [52].

Presence of bacteria has previously been reported in the circulation and atherosclerotic plaques of CVD patients [8], [53]. Moreover, of late, presence of viruses has been reported in the circulation of healthy individuals [27]. Nevertheless, viruses have not been reported in the circulation of CVD patients until now. In CVD patients, cardiomyocyte death (apoptosis or necrosis) might be induced by inflammatory response to microbial or viral infection. Inflammation and oxidative stress, exacerbated by infection could play a critical role in the pathogenesis and destabilization of blood vessels in CAD resulting in ischemia. Following ischemia, reactive oxygen species (ROS) induce DNA single strand breakage and subsequent activation of the poly ADP ribose-synthetase causes necrotic cell death [54]. The cellular components from the necrotic cardiomyocytes including DNA might be released into the peripheral circulation.

The major source of circulating bacterial DNA could be microbial translocation. Microbial/bacterial translocation refers to the passage of viable inhabiting bacteria and molecules like endotoxins and lipopolysaccharides, across the intestinal barrier to the blood. Among CVD patients, microbial translocation occurs more frequently in patients with chronic heart failure (CHF) because of intestinal edema followed by dysfunction of gut immunity. It was earlier reported that intestinal barrier dysfunction with an increased paracellular permeability, and an augmented intestinal bacterial biofilm are present in CHF patients [55]. Similarly, bacterial translocation from the GI tract, in particular, from oral cavity to the atherosclerotic lesions of CAD patients [53], [56]. More recently, from studies on the human virome, it is suspected that presence of viruses in asymptomatic humans is primarily due to viral translocation, a concept not worked upon extensively, until now [57].

Cardiac failure and cardiac surgeries could contribute to the large differences observed in circulating DNA between test and control. Surgical treatment in most of the patients may result in local and systemic inflammation leading to exacerbated gastrointestinal hypoperfusion, increased bowel permeability, increased endotoxemia and elevated levels of cytokines, thereby causing conditions like Acute Bowel ischemia (ABI), Ischemia-reperfusion (IR) injury, multiple organ dysfunction syndrome (MODS). In particular, the inflammatory response after vascular Ischemia-reperfusion (IR) injury has been known to result in systemic inflammatory response syndrome (SIRS) or multi-organ dysfunction syndrome (MODS). It has already been reported that cardiac surgeries increase intestinal permeability leading to gut ischemia resulting in the translocation of microorganisms and endotoxins into the systemic circulation through the portal and lymphatic systems [58], leading to a continuing toxic insult and systemic tissue injury. In addition, surgical trauma itself contributes to the complex inflammatory response that occurs after cardiac procedures [59]. Metformin treatment was given only for two coronary artery disease (CAD) patients. Both these patients (CVD031 and CVD077) had comparatively higher circDNA levels, 16S rRNA copy number and β-globin gene copy number. However, metformin treatment does not seem to impact the circulating DNA levels by a large margin considering that only 2 patients were given this drug.

This is the first study to report the relative levels of bacterial elements and human DNA in the circulation of CVD patients. Recently, it has been reported that dysbiosis in blood microbiota is associated with the onset of cardiovascular events. The proteobacterial concentration in blood was positively correlated with the onset of cardiovascular complications [8]. Since these authors have used whole blood DNA for quantitative PCR, they have not distinguished the presence of bacterial cells or cell-free DNA in circulation. In the present study, bacterial 16S rRNA copy number was significantly higher in the plasma, implying the increased presence of cell-free bacterial DNA elements in the circulation of CVD patients. Moreover, the ratio of 16S rRNA/β-globin gene copy number was significantly higher in CVD patients. This shows a probable diagnostic value in discriminating CVD patients from healthy controls with good sensitivity and specificity.

Also, the β-globin gene copy number was significantly higher in the plasma of CVD patients. β-globin gene is normally used as a marker of circulating DNA and its copy number will be increased when there is trauma or injury to the internal organs, indicating an increase in the concentration of circulating DNA. The up-regulation of β-globin gene in the circulation of CVD patients has been reported in earlier studies [60]. The up-regulation of β-globin gene has been reported to have prognostic value in CVDs. In particular, the extent of tissue damage and correspondingly, the clinical outcome, in cases like myocardial infarction (MI), coronary artery disease (CAD) and chest pain patients were found to be directly correlated with the concentration of circulating DNA, as detected by the increase in β-globin gene copy number in these patients. Additionally, in the present study, we have found an increased 16S rRNA gene copy number proportional to the increased β -globin gene copy number in CVD patients.

The total sequence throughput for the test samples was 183 Mb (3 samples) whereas that for the control samples was only 74 Mb (3 samples). The sequence throughput for each sample was too low, in the range of 22 to 27 Mb for control samples and 32 to 96 Mb for test samples. Similar results were obtained in the study by Li et al (2011), where the total sequence throughput for the test samples (n = 10) was 658 Mb and that for the control samples (n = 10) was 410 Mb [36]. The sequence throughput was low in all the samples because the circulating DNA concentration will be normally meager in physiologically healthy individuals. This concentration might increase in pathological conditions like CVDs or HIV/AIDS. As can be seen from the results obtained in the present study, the sequence throughput obtained for the test samples was nearly 2.5 fold higher than that obtained for the control samples.

Earlier, we have reported a higher Proteobacterial abundance in CVD samples by amplified 16S rRNA gene sequence analysis [7]. Paradoxically, in the present study, by shotgun sequencing of human plasma, we found that CVD samples had an increased Actinobacterial count than Proteobacterial count in comparison with healthy individuals. Nonetheless, both Actinobacteria and Proteobacteria were the predominant phyla in CVD patients and healthy individuals. By comparing metagenomic shotgun sequencing and 16S rRNA based amplicon sequencing results (aforementioned study) of these six samples individually, we observed dissimilar findings. Among the three control samples, we observed higher levels of Proteobacteria and Firmicutes by amplicon sequencing. In contrast, higher levels of Proteobacteria and very low levels of Firmicutes were observed in the same samples by shotgun sequencing. Among the three CVD samples, we observed phylum Proteobacteria to be dominant, occupying more than 95% by amplicon sequencing. In shotgun sequence analysis, Actinobacterial sequences were higher in the same samples. Overall, Proteobacteria, Actinobacteria and Firmicutes formed a major fraction in all the six samples. Further, from amplicon sequencing results, it was interesting to note that Actinobacteria was the next predominant phylum after Proteobacteria among CVD samples and after Proteobacteria and Firmicutes among control samples.

The difference in outcomes between the aforementioned and the present study could be attributed to the method of sequencing employed in both the studies. In our earlier amplicon sequencing study, the 16S rRNA primers used encompasses the V3 hyper variable region of the 16S rRNA gene. Although hypervariable regions V2, V3 and V6 were found to provide maximum discriminating power for studying bacterial diversity, no single hypervariable region of the 16S rRNA gene has been confirmed to distinguish the entire prokaryotic kingdom [61]. Therefore, short range 16S rRNA primers targeting a single variable region of 16S rRNA gene may be biased due to unequal amplification of 16S rRNA genes and may miss some key members of the microbial communities [62].

On the other hand, shotgun sequencing gives complete profiling of the microbial communities inhabiting a particular site without any amplification bias. Further, by screening the virome, we found that sequences from CVD plasma had a larger range of viral species compared to sequences from healthy plasma. Bacteriophages were dominant among CVD samples while eukaryotic viral elements were dominant among control samples. Remarkably, similar findings were reported in a recent study [36], where bacteriophages and retroviral elements were dominant in the circulation of HIV/AIDS patients in contrast to the dominance of eukaryotic anelloviral elements in the circulation of healthy individuals [36]. However, eukaryotic viral elements were found in both diseased and healthy plasma in both these studies.

Investigation of the circulating virome in CVD patients has uncovered a myriad of resident phage sequences associated with their respective host organisms. Recent evidence claims that the blood in apparently healthy persons is not sterile and may contain many viral species composed majorly of ssDNA viruses belonging to the family Anelloviridae with TTVs being the most frequently identified [27], [36]. When samples from large enough human cohorts are analyzed, it will be feasible to determine viral prevalence, likely routes of transmission, and most crucially, its association with CVDs.

In the present study, many genes of clinical significance, which could be involved in pathogenesis, have been identified in the circulation of CVD patients. In particular, antibiotic resistance genes, virulence genes, toxin genes and genes involved in secretory pathways have been identified in the circulating microbiome of both CVD patients and healthy individuals. More specifically, the number of clinically significant genes identified was higher in CVD patients (Tables S4 and S5 in Supporting Information S1). Since majority of viral elements found in the circulation of CVD patients are bacteriophages, which are known to transfer genes between microbial hosts, there is a potential for exchange of antibiotic resistance genes among bacteria.

Bacteriophages are believed to play an influential role in shaping the development and functional outputs of host microbiomes. Phages can influence processes such as bacterial virulence and pathogenesis through their effects on host bacteria and can contribute genes that are beneficial to their bacterial hosts which in turn assists their own survival and propagation [63]. This gene flow suggests that phages may have an influential role in the adaptation of the microbiome to stressful environments and to evade the host immune system.

We attempted to profile CVD specific circulating microbiomes. Despite small changes in the circulating microbiome (Fig 7, 8); all three disease samples displayed a high Actinobacteria- Proteobacteria ratio and bacteriophages-eukaryotic viral elements ratio. Congenital (CHD), valvular (VHD) and ischemic heart disease (IHD) group samples were grouped together into a single cohort while drawing inferences from the deep sequencing data since only three CVD samples were taken up for the analysis comprising, one each from all the three groups. Therefore, these samples were not analyzed individually, although, the samples were from patients with different disease etiology. Moreover, the major and significant observations made with respect to both bacteriome and virome compositions were similar in all the three disease types leading to a generalized conclusion. This deep sequencing analysis is a preliminary study and the first study of this kind. Therefore, the conclusion drawn from this study needs to be affirmed further by studies involving larger number of samples.

Though cancer cell DNaemia [11] and bacteremia in sepsis conditions [13] have been reported previously, the occurrence of ‘bacterial DNaemia’ and ‘viral DNaemia’, and its impact on human health needs to be explored in view of our microbial inhabitants. While oral, skin, gut, dental and trauma induced entry routes have been suggested for microbial ingress; further research will enable us to examine the role of these bacterial and viral elements in the possible onset or progression of CVDs.

As a proof of principle, this study presents the value of coupling metagenomics with clinical findings, helping us to move closer to molecular diagnosis. The primary significance of this study is the combined use of metagenomic sequencing and clinical microbiology for understanding the circulating microbiome in CVD patients. The limitations of this study include the lesser number of samples analysed and low sequencing depth obtained in the tested samples. Therefore, further studies by increasing the sample size and sequencing depth is necessary to understand the importance of microbiome in the molecular diagnosis of CVDs. The CVD samples showed an increased count of actinobacteria and bacteriophages in comparison with healthy individuals. Further exploration of the dominance and suppression of selected bacterial and viral populations in the human circulation, on a large scale, is essential before one can conclude if this perturbation is a cause or effect of CVD incidence.

## Conclusion

We have shown that circDNA concentration is elevated in CVD patients. The presence of bacterial and viral DNA fragments also contributed to the circDNA concentration. The elevated circDNA levels and the higher ratio of 16S rRNA/β-globin genes were observed in CVD patients. In addition, we found a higher circulating microbial diversity in CVD patients in comparison to healthy individuals.

## Supporting Information

Supporting Information S1 **Combined supporting information file.** Table S1. Baseline characteristics of CVD patients and healthy individuals used in this study. Table S2. Primers used in real-time PCR. Table S3. Baseline characteristics of CVD patients and healthy individuals used for Plasma DNA Sequencing. Table S4. COGs involved in pathogenesis identified from the circulating microbiome of CVD patients. Table S5. COGs involved in virulence pathways identified from the circulating microbiome of healthy individuals.
(DOC)
