# Supplementary material for: Elevated Levels of Circulating DNA in Cardiovascular Disease Patients: Metagenomic Profiling of Microbiome in the Circulation
Source: PLoS One. 2014 Aug 18;9(8):e105221. doi: 10.1371/journal.pone.0105221 (PMC4136842; doi:10.1371/journal.pone.0105221)
Supplement: Supporting Information S1 — Combined supporting information file. Table S1. Baseline characteristics of CVD patients and healthy individuals used in this study. Table S2. Primers used in real-time PCR. Table S3. Baseline characteristics of CVD patients and healthy individuals used for Plasma DNA Sequencing. Table S4. COGs involved in pathogenesis identified from the circulating microbiome of CVD patients. Table S5. COGs involved in virulence pathways identified from the circulating microbiome of healthy individuals. (DOC) [file pone.0105221.s001.doc]

**Table S1. Baseline characteristics of CVD patients and healthy individuals used in this study**

| **Sl. No.** | **Characteristics** | **CVD Patients**  **(n=80)** | **VHD**  **(n=24)** | **IHD**  **(n=30)** | **CHD**  **(n=26)** | **Control**  **(n=40)** |
| --- | --- | --- | --- | --- | --- | --- |
| 1 | Age | 29.6 ± 14.3 | 29.2 ± 11.4 | 51.3 ± 9 | 21.9 ± 10.4 | 32.6 ± 9 |
| 2 | Sex (Male: Female) | 49:31 | 9:15 | 27:3 | 13:13 | 33:7 |
| **Cardiovascular Risk Factors No. (%)** | | | | | | |
| 3 | Obesity | 13 (16.25) | 2 (8.33) | 8 (26.66) | 3 (11.5) | 0 (0) |
| 4 | Arterial Hypertension | 4 (5) | 0 (0) | 3 (10) | 1 (3.85) | 1 (2.5) |
| 5 | Diabetes mellitus | 13 (16.25) | 3 (12.5) | 8 (26.66) | 2 (7.7) | 1 (2.5) |
| 6 | Smoking/Tobacco | 27 (33.75) | 4 (16.66) | 21 (70) | 2 (7.7) | 26 (65) |
| 7 | Alcohol | 23 (28.75) | 4 (16.66) | 17 (56.66) | 2 (7.7) | 26 (65) |
| 8 | Genetic Background (Presence of CVDs in parents/other relations) | 10 (12.5) | 0 (0) | 7 (28.33) | 3 (11.5) | 0 (0) |
| 9 | Without any known risk factors | 32 (40) | 13 (54.16) | 2 (6.66) | 17 (65.4) | 12 (30) |

**Table S2. Sequence information of primers for real-time PCR**

| **Gene** | **Primers (5’-3’)** | **Product size (bp)** | **Reference** |
| --- | --- | --- | --- |
| 16S rRNA | F:TCCTACGGGAGGCAGCAGT  R:TTACCGCGGCTGCTGGCAC | 202 | This study |
| β-globin | F:GTGCACCTGACTCCTGAGGAGA  R:CCTTGATACCAACCTGCCCAG | 101 | [41] |

**Table S3. Baseline characteristics of CVD patients and healthy individuals used for Plasma DNA Sequencing**

| **Sl.**  **No.** | **Sample ID** | **Age** | **Sex** | **Type** | **Disease Status** |
| --- | --- | --- | --- | --- | --- |
| 1. | CVD010 | 13 | M | Test | Partial anomalous pulmonary venous connection |
| 2. | CVD008 | 44 | M | Test | Bicuspid aortic valvular disease |
| 3. | CVD014 | 42 | M | Test | Coronary artery disease |
| 4. | CON064 | 31 | M | Control | - |
| 5. | CON029 | 25 | M | Control | - |
| 6. | CON030 | 25 | F | Control | - |

**Table S4. COGs involved in pathogenesis identified from the circulating microbiome of CVD patients**

| **Sl. No.** | **Sample ID** | **COG No.** | **Gene function annotation** | **No. of genes** | **Function** |
| --- | --- | --- | --- | --- | --- |
|  | CVD010 | COG0595 | Predicted hydrolase of the metallo-beta-lactamase superfamily | 1 | General function prediction only |
|  | CVD010 | COG0841 | Cation/multidrug efflux pump | 19 | Defense mechanisms |
|  | CVD010 | COG1131 | ABC-type multidrug transport system, ATPase component | 11 | Defense mechanisms |
|  | CVD010 | COG1132 | ABC-type multidrug transport system, ATPase and permease components | 5 | Defense mechanisms |
|  | CVD010 | COG1234 | Metal-dependent hydrolases of the beta-lactamase superfamily III | 1 | General function prediction only |
|  | CVD010 | COG1253 | Hemolysins and related proteins containing CBS domains | 6 | General function prediction only |
|  | CVD010 | COG1450 | Type-II secretory pathway, component *Pul*D | 2 | Multiple classes |
|  | CVD010 | COG1523 | Type-II secretory pathway, pullulanase *Pul*A and related glycosidases | 9 | Carbohydrate transport and metabolism |
|  | CVD010 | COG1566 | Multidrug resistance efflux pump | 2 | Defense mechanisms |
|  | CVD010 | COG1680 | Beta-lactamase class C and other penicillin binding proteins | 7 | Defense mechanisms |
|  | CVD010 | COG2333 | Predicted hydrolase (metallo-beta-lactamase superfamily) | 2 | General function prediction only |
|  | CVD010 | COG2367 | Beta-lactamase class A | 37 | Defense mechanisms |
|  | CVD010 | COG2948 | Type-IV secretory pathway, *Vir*B10 components | 29 | Intracellular trafficking, secretion, and vesicular transport |
|  | CVD010 | COG3267 | Type-II secretory pathway, component *Exe*A (predicted ATPase) | 1 | Intracellular trafficking, secretion, and vesicular transport |
|  | CVD010 | COG4452 | Inner membrane protein involved in colicin E2 resistance | 1 | Defense mechanisms |
|  | CVD010 | COG0534 | Na^+^-driven multidrug efflux pump | 1 | Defense mechanisms |
|  | CVD010 | COG0861 | Membrane protein *Ter*C, possibly involved in tellurium resistance | 3 | Inorganic ion transport and metabolism |
|  | CVD010 | COG4796 | Type-II secretory pathway, component *Hof*Q | 1 | Intracellular trafficking, secretion, and vesicular transport |
|  | CVD014 | COG0534 | Na^+^-driven multidrug efflux pump | 4 | Defense mechanisms |
|  | CVD014 | COG0595 | Predicted hydrolase of the metallo-beta-lactamase superfamily | 7 | General function prediction only |
|  | CVD014 | COG0841 | Cation/multidrug efflux pump | 4 | Defense mechanisms |
|  | CVD014 | COG1131 | ABC-type multidrug transport system, ATPase component | 13 | Defense mechanisms |
|  | CVD014 | COG1132 | ABC-type multidrug transport system, ATPase and permease components | 7 | Defense mechanisms |
|  | CVD014 | COG1234 | Metal-dependent hydrolases of the beta-lactamase superfamily III | 2 | General function prediction only |
|  | CVD014 | COG1237 | Metal-dependent hydrolases of the beta-lactamase superfamily II | 1 | General function prediction only |
|  | CVD014 | COG1253 | Hemolysins and related proteins containing CBS domains | 2 | General function prediction only |
|  | CVD014 | COG1459 | Type-II secretory pathway, component *Pul*F | 2 | Multiple classes |
|  | CVD014 | COG1968 | Uncharacterized bacitracin resistance protein | 2 | Defense mechanisms |
|  | CVD014 | COG2333 | Predicted hydrolase (metallo-beta-lactamase superfamily) | 2 | General function prediction only |
|  | CVD014 | COG2348 | Uncharacterized protein involved in methicillin resistance | 1 | Defense mechanisms |
|  | CVD014 | COG2367 | Beta-lactamase class A | 10 | Defense mechanisms |
|  | CVD014 | COG2804 | Type-II secretory pathway, ATPase *Pul*E/TFP pilus assembly pathway, ATPase *Pil*B | 1 | Multiple classes |
|  | CVD014 | COG3505 | Type-IV secretory pathway, *Vir*D4 components | 1 | Intracellular trafficking, secretion, and vesicular transport |
|  | CVD014 | COG4452 | Inner membrane protein involved in colicin E2 resistance | 2 | Defense mechanisms |
|  | CVD014 | COG4796 | Type-II secretory pathway, component *Hof*Q | 1 | Intracellular trafficking, secretion, and vesicular transport |
|  | CVD014 | COG1523 | Type-II secretory pathway, pullulanase *Pul*A and related glycosidases | 2 | Carbohydrate transport and metabolism |
|  | CVD008 | COG1680 | Beta-lactamase class C and other penicillin binding proteins | 3 | Defense mechanisms |
|  | CVD008 | COG2333 | Predicted hydrolase (metallo-beta-lactamase superfamily) | 4 | General function prediction only |
|  | CVD008 | COG1132 | ABC-type multidrug transport system, ATPase and permease components | 45 | Defense mechanisms |
|  | CVD008 | COG1234 | Metal-dependent hydrolases of the beta-lactamase superfamily III | 5 | General function prediction only |
|  | CVD008 | COG1235 | Metal-dependent hydrolases of the beta-lactamase superfamily I | 1 | General function prediction only |
|  | CVD008 | COG0630 | Type-IV secretory pathway, *Vir*B11 components, and related ATPases involved in archaeal flagella biosynthesis | 31 | Multiple classes |
|  | CVD008 | COG0842 | ABC-type multidrug transport system, permease component | 1 | Defense mechanisms |
|  | CVD008 | COG1237 | Metal-dependent hydrolases of the beta-lactamase superfamily II | 2 | General function prediction only |
|  | CVD008 | COG1668 | ABC-type Na^+^ efflux pump, permease component | 1 | Multiple classes |
|  | CVD008 | COG2348 | Uncharacterized protein involved in methicillin resistance | 7 | Defense mechanisms |
|  | CVD008 | COG2720 | Uncharacterized Vancomycin resistance protein | 2 | Defense mechanisms |
|  | CVD008 | COG2804 | Type-II secretory pathway, ATPase *Pul*E/TFP pilus assembly pathway, ATPase *Pil*B | 5 | Multiple classes |
|  | CVD008 | COG3451 | Type-IV secretory pathway,  *Vir*B4 components | 125 | Intracellular trafficking, secretion, vesicular transport |
|  | CVD008 | COG3504 | Type-IV secretory pathway,  *Vir*B9 components | 29 | Intracellular trafficking, secretion, and vesicular transport |
|  | CVD008 | COG3702 | Type-IV secretory pathway,  *Vir*B3 components | 8 | Intracellular trafficking, secretion, and vesicular transport |
|  | CVD008 | COG3704 | Type-IV secretory pathway,  *Vir*B6 components | 16 | Intracellular trafficking, secretion, and vesicular transport |
|  | CVD008 | COG3736 | Type-IV secretory pathway, component *Vir*B8 | 51 | Intracellular trafficking, secretion, and vesicular transport |
|  | CVD008 | COG3843 | Type-IV secretory pathway, *Vir*D2 components (relaxase) | 1 | Intracellular trafficking, secretion, and vesicular transport |
|  | CVD008 | COG4531 | ABC-type Zn^2+^ transport system, periplasmic component/surface adhesin | 2 | Inorganic ion transport and metabolism |
|  | CVD008 | COG1131 | ABC-type multidrug transport system, ATPase component | 77 | Defense mechanisms |
|  | CVD008 | COG0841 | Cation/multidrug efflux pump | 8 | Defense mechanisms |
|  | CVD008 | COG1523 | Type-II secretory pathway, pullulanase *Pul*A and related glycosidases | 17 | Carbohydrate transport and metabolism |
|  | CVD008 | COG2274 | ABC-type bacteriocin/lantibiotic exporters, contain an N-terminal double-glycine peptidase domain | 68 | Defense mechanisms |
|  | CVD008 | COG2367 | Beta-lactamase class A | 11 | Defense mechanisms |

**Table S5. COGs involved in virulence pathways identified from the circulating microbiome of healthy individuals**

| **Sl. No.** | | **Sample ID** | | **COG No.** | | **Gene function annotation** | **No. of genes** | **Function** |
| --- | --- | --- | --- | --- | --- | --- | --- | --- |
| 1. | | CON029 | | COG1234 | | Metal-dependent hydrolases of the beta-lactamase superfamily III | 1 | General function prediction only |
| 2. | | CON029 | | COG4531 | | ABC-type Zn^2+^ transport system, periplasmiccomponent/surface adhesin | 3 | Inorganic ion transport and metabolism |
| 3. | | CON064 | | COG0630 | | Type-IV secretory pathway, *Vir*B11 components, and related ATPases involved in archaeal flagella biosynthesis | 1 | Multiple classes |
| 4. | | CON064 | | COG1131 | | ABC-type multidrug transport system, ATPase component | 2 | Defense mechanisms |
| 5. | | CON064 | | COG1132 | | ABC-type multidrug transport system, ATPase and permease components | 1 | Defense mechanisms |
| 6. | | CON064 | | COG1237 | | Metal-dependent hydrolases of the beta-lactamase superfamily II | 1 | General function prediction only |
| 7. | | CON064 | | COG1523 | | Type-II secretory pathway, pullulanase *Pul*A and related glycosidases | 1 | Carbohydrate transport and metabolism |
| 8. | | CON064 | | COG1968 | | Uncharacterized bacitracin resistance protein | 1 | Defense mechanisms |
| 9. | | CON064 | | COG2274 | | ABC-type bacteriocin/lantibiotic exporters, contain an N-terminal double-glycine peptidase domain | 2 | Defense mechanisms |
| 10. | | CON064 | | COG2367 | | Beta-lactamase class A | 331 | Defense mechanisms |
| 11. | | CON064 | | COG2804 | | Type-II secretory pathway, ATPase *Pul*E/TFP pilus assembly pathway, ATPase *Pil*B | 1 | Multiple classes |
| 12. | | CON064 | | COG3309 | | Uncharacterized virulence-associated protein D | 2 | Function unknown |
| 13. | | CON030 | | COG0841 | | Cation/multidrug efflux pump | 1 | Defense mechanisms |
| 14. | | CON030 | | COG2367 | | Beta-lactamase class A | 128 | Defense mechanisms |
| 15. | | CON030 | | COG1131 | | ABC-type multidrug transport system, ATPase component | 1 | Defense mechanisms |
|  |  | |  | |  | | | |
